# Supplementary material for: Optimal government and manufacturer incentive contracts for green production with asymmetric information
Source: PLoS One. 2023 Aug 9;18(8):e0289639. doi: 10.1371/journal.pone.0289639 (PMC10411796; doi:10.1371/journal.pone.0289639)
Supplement: S1 Table — (DOCX) [file pone.0289639.s001.docx]

**Table S1.** Summary of notations

| Symbol | Description |
| --- | --- |
| **Parameters** |  |
| *g* | Green-degree of a product, $g\in[\underline{g},\bar{g}]$, $0\leq\underline{g},\bar{g}\leq1$ |
| *a* | Base market capacity of products, *a*>0 |
| *b* | The price elasticity of product demand coefficient, *b*>0 |
| *h* | Attraction coefficient of the green product, *h*>0 |
| *a*_1_, *a*_2_, *a*_3_ | $a_{1}=a/b$, $a_{2}=1/b$, $a_{3}=h/b$, $a_{1},a_{2},a_{3}>0$ |
| $\underline{c}$ | The unit cost of a conventional product, $\underline{c}>0$ |
| *z* | The extra cost factor of green product production, *z*>0 |
| *v* | The coefficient of environmental benefit per unit of green product, *v*>0 |
| $\alpha$ | The fixed ratio of benefit-sharing, $\alpha>0$ |
| $\mu$ | The flexible coefficient of benefit-sharing, $\mu>0$ |
| $\underline{u}_{i}$ | Member *i*'s utility at the start of negotiation in the Nash bargaining model, $i=1,\cdots,n$ |
| $\gamma_{i}$ | The bargaining power of member *i* in the Nash bargaining model, $\sum_{i=1}^{n} \gamma_{i}=1$, $i=1,\cdots,n$ |
| **Variables & Functions** |  |
| *q* | Governmental green procurement volume, which equals the manufacturer production output of green products, $q=a-bp+hg^{2}$ |
| $t$ | The lump-transfer payment from government to the manufacturer |
| $f\left(g\right)$ | The probability density function of green-degree |
| $F\left(g\right)$ | The cumulative distribution function of green-degree |
| *p* | Unit procurement price of the green product |
| $\pi_{M}$ | Manufacturer's profit for producing green products |
| $U_{G}$ | Government's benefit from the green products |
| $\pi_{T}$ | Total benefits of manufacturer and government |
| $u_{i}$ | The utility function of member *i* in the Nash bargaining model, $i=1,\cdots,n$ |
