# Supplementary material for: Optimal government and manufacturer incentive contracts for green production with asymmetric information
Source: PLoS One. 2023 Aug 9;18(8):e0289639. doi: 10.1371/journal.pone.0289639 (PMC10411796; doi:10.1371/journal.pone.0289639)
Supplement: S1 Fig — (DOCX) [file pone.0289639.s002.docx]

**S2 Fig. Consumer surplus (CS)**

The government in our research is a consumer that purchases products from manufacturers. Consumer surplus refers to the difference between the highest price that the government is willing to pay and the actual price paid. Using the green product demand function $q(g)=a-bp(g)+hg^{2}$ and the price function $p(g)=a_{1}-a_{2}q(g)+a_{3}g^{2}$, we obtain the relationship between green product's procurement price and its demand for the government. The functions also reflect the change in consumer surplus due to a quantity change. See Fig S2. The shaded area represents the consumer surplus $CS=q(a_{1}+a_{3}g^{2}-a_{1}+a_{2}q-a_{3}g^{2})/2=a_{2}q^{2}/2$.


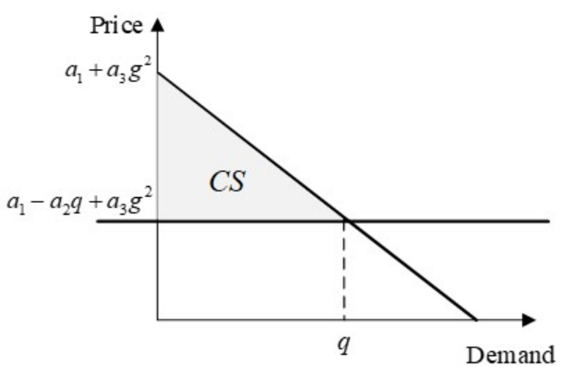


**Fig S2. Influence of change in demand on consumer surplus**
