# Supplementary material for: Optimal government and manufacturer incentive contracts for green production with asymmetric information
Source: PLoS One. 2023 Aug 9;18(8):e0289639. doi: 10.1371/journal.pone.0289639 (PMC10411796; doi:10.1371/journal.pone.0289639)
Supplement: S1 Appendix — (DOCX) [file pone.0289639.s003.docx]

**S3 Appendix Equivalent conversion of programming problem (P1)**

According to the revelation principle, for a manufacturer of type *g*, the government should design a contract that meets the condition:

$\left[a_{1}-a_{2}q(g)+(a_{3}-0.5z)g^{2}-\underline{c}\right]q(g)+t(g)\geq\left[a_{1}-a_{2}q(\tilde{g})+(a_{3}-0.5z)g^{2}-\underline{c}\right]q(\tilde{g})+t(\tilde{g})$ (C-1)

that is, manufacturers of type *g* cannot counterfeit into type $\tilde{g}$ for a contract of type $\tilde{g}$. Based on Eq. (C-1), $\forall\left(g,g'\right)\in G$, we have

$\left[a_{1}-a_{2}q(g)+(a_{3}-0.5z)g^{2}-\underline{c}\right]q(g)+t(g)\geq\left[a_{1}-a_{2}q(\tilde{g})+(a_{3}-0.5z)g^{2}-\underline{c}\right]q(\tilde{g})+t(\tilde{g})$ (C-2)

$\left[a_{1}-a_{2}q(g')+(a_{3}-0.5z)g'^{2}-\underline{c}\right]q(g')+t(g')\geq\left[a_{1}-a_{2}q(g)+(a_{3}-0.5z)g'^{2}-\underline{c}\right]q(g)+t(g)$ (C-3)

The sum of Eq. (C-2) and Eq. (C-3) is given by

$(a_{3}-0.5z)(g+g')(g-g')\left[q(g)-q(g')\right]\geq0$ (C-4)

From Eq. (C-4), when $\alpha\geq a_{3}-0.5z$, for any type $g\in G$, $q(g)$ increases with *g* and can be differentiated in the interval $[\underline{g},\bar{g}]$ (except on$\underline{g}$and$\bar{g}$), that is

(C-5)

Besides, the response function $\tilde{g}$ to g derived from Eq. (C-1) is

(C-6)

According to the direct revelation mechanism, for all $g\in G$, we have

(C-7)

Then the first-order derivative of $\pi_{M}\left(g\right)$ is expressed as follows

$=2(a_{3}-0.5z)gq(g)$ (C-8)
