# Supplementary material for: Optimal government and manufacturer incentive contracts for green production with asymmetric information
Source: PLoS One. 2023 Aug 9;18(8):e0289639. doi: 10.1371/journal.pone.0289639 (PMC10411796; doi:10.1371/journal.pone.0289639)
Supplement: S2 Appendix — (DOCX) [file pone.0289639.s004.docx]

**S4 Appendix The optimal incentive contract under symmetric information**

To ensure the accomplishment of agent tasks by the manufacturers, the government should ensure their profit at least $\underline{\pi}_{M}$. The design of the incentive mechanism under asymmetric information is expressed as a programming problem (P4), shown as follows.

(P4): $\mathop{\max EU_{G}}\limits_{\{t(g),q(g)\}} =(vg-\underline{c}-0.5zg^{2})q(g)+a_{2}q^{2}(g)/2-t(g)$

*s.t.* $\pi_{M}(g)=[a_{1}-a_{2}q(g)+(a_{3}-0.5z)g^{2}-\underline{c}]q(g)+t(g)\geq\underline{\pi}_{M}$ (D-1)

Eq. (D-1) is the manufacturer's IR constraint. Under complete information, we assume $\pi_{M}=\underline{\pi}_{M}$, then the transfer payment obtained by manufacturers satisfies

$t(g)=\underline{\pi}_{M}-[a_{1}-a_{2}q(g)+(a_{3}-0.5z)g^{2}-\underline{c}]q(g)$ (D-2)

Plugging Eq. (D-2) into the objective function in (P4), we get

$\mathop{\max EU_{G}}\limits_{\{q(g)\}} =(vg-\underline{c}-0.5zg^{2})q(g)+a_{2}q^{2}(g)/2+[a_{1}-a_{2}q(g)+(a_{3}-0.5z)g^{2}-\underline{c})q(g)-\underline{\pi}_{M}$ (D-3)

Maximizing Eq. (D-3) yields the first-order condition for *q*, $\partial EU_{G}/\partial q=0$, and the second-order derivative $\partial^{2}EU_{G}/\partial q^{2}=-a_{2}<0$. We get the optimal output of green products $q^{*}\left(g\right)$, presented in Eq. (31). Combining with Eq. (D-2), we get $t^{*}\left(g\right)$, shown in Eq. (32).
