# Supplementary material for: Optimal government and manufacturer incentive contracts for green production with asymmetric information
Source: PLoS One. 2023 Aug 9;18(8):e0289639. doi: 10.1371/journal.pone.0289639 (PMC10411796; doi:10.1371/journal.pone.0289639)
Supplement: S3 Appendix — (DOCX) [file pone.0289639.s005.docx]

**S5 Appendix Solution of transfer payment in the Nash bargaining model**

Let $M=(vg-\underline{c}-0.5zg^{2})q^{*}(g)+a_{2}[q^{*}(g)/2]^{2}-U_{G,(3)}^{SB}(g)$ and $N=(a_{1}-a_{2}q(g)+(a_{3}-0.5z)g^{2}-\underline{c})q^{*}(g)-\pi_{M,(3)}^{SB}(g)$. $U_{G,(3)}^{SB}(g)$ and $\pi_{M,(3)}^{SB}(g)$ are considered fixed. Combined with Eqs. (36)-(38), we get Eq. (E-1) and calculate its first-order derivative to *t*.

$U\left(t\right)=\left(M-t\right)^{1-\gamma}\left(N+t\right)^{\gamma}$ (E-1)

$\partial U/\partial t=\left(M-t\right)^{-\gamma}\left(N+t\right)^{1-\lambda}\left[\gamma M+\left(\gamma-1\right)N-t\right]=0$ (E-2)

It requires $M-t\geq0$ and $N+t\geq0$ under Pareto improvement of contractors' profits. So the second-order derivative of $U\left(t\right)$ is expressed as

$\partial^{2}U/\partial t^{2}=-\gamma\left(1-\gamma\right)\left(M-t\right)^{-\gamma-1}\left(N+t\right)^{\gamma-2}\left(M+N\right)^{2}\leq0$ (E-3)

From Eq. (E-1), we obtain $t^{NB}\left(g\right)$, shown in Eq. (39).
