# Supplementary material for: Optimal government and manufacturer incentive contracts for green production with asymmetric information
Source: PLoS One. 2023 Aug 9;18(8):e0289639. doi: 10.1371/journal.pone.0289639 (PMC10411796; doi:10.1371/journal.pone.0289639)
Supplement: S4 Appendix — (DOCX) [file pone.0289639.s006.docx]

**S6 Appendix** **Explanation for revealing the true green-degree for Company X**

According to the revelation principle, we have

$\left[a_{1}-a_{2}q(g)+\left(a_{3}-0.5z\text{+}\mu v\right)g^{2}-\underline{c}\right]q(g)+t(g)\geq\left[a_{1}-a_{2}q(g')+\left(a_{3}-0.5z\text{+}\mu v\right)g^{2}-\underline{c}\right]q(g')+t(g')$ (F-1)

where *g* is the true green-degree of the company. The company reports a counterfeit greed-degree $g'$.

Company X's profit can be obtained by substituting value from Table 1 and the parameters used in Section 8 in:

$\pi_{M}(g')=\left[a_{1}-a_{2}q(g')+\left(a_{3}-0.5z\text{+}\mu v\right)g^{2}-\underline{c}\right]q(g')+t(g')$ (F-2)

Thus, the manufacturer's profits when reporting a false value of green-degree are shown in Table S6.

**Table S6. Manufacturer's profit in reporting a false value of *g***

| $g'$ | 0.25 | 0.3 | 0.35* | 0.4 | 0.45 | 0.5 |
| --- | --- | --- | --- | --- | --- | --- |
| ($) | 542 | 571 | 610 | 588 | 601 | 608 |

*Note that the actual value of *g* is 0.35.
